# Hydrogen and Propane Production From Butyric Acid Photoreforming Over Pt-TiO_2_

**DOI:** 10.3389/fchem.2019.00563

**Published:** 2019-08-07

**Authors:** Gabriele Scandura, Jorge Rodríguez, Giovanni Palmisano

**Affiliations:** ^1^Department of Chemical Engineering, Masdar Institute Campus, Khalifa University, Abu Dhabi, United Arab Emirates; ^2^Research and Innovation Center on CO_2_ and H_2_ (RICH), Khalifa University, Abu Dhabi, United Arab Emirates

**Keywords:** hydrogen, propane, photoreforming, butyric acid, titanium dioxide, reaction mechanism

## Abstract

Photocatalysis is a promising technology from economic, energetic, and ecological points of view because it takes advantage of solar light. Hence, it is one of the investigated green routes to produce hydrogen from renewable energy resources. Butyric acid (BA) is largely present in wastewater and as an intermediate product in anaerobic digestion and therefore it is an inexpensive resource, which can be converted to valuable chemicals. In this work, photoreforming of butyric acid (BAPR) under UV light in aqueous suspensions of platinum-modified titanium dioxide-based catalysts is reported for the first time. Titania nanotubes (TNT) synthesized and calcined at different temperatures (300, 400, 500°C) and commercial TiO_2_ (P25), decorated with platinum nanoparticles, have been tested and characterized through different techniques including X-ray powder diffraction, UV-vis diffuse reflectance and photoluminescence spectroscopy, transmission electron microscopy, BET and porosimetry analysis. The main identified products of the BAPR were H_2_, propane, CO_2_ and several organic acids (e.g., pentanoic and 3-methylhexanoic acid). It has been found that the morphology and crystallinity of the photocatalysts affected dramatically their optical properties and, consequently, the reaction rate and the product distribution. Specifically, the highest conversion of BA (X_BA_) and selectivity toward H_2_ (S_H2_) was recorded with P25-Pt (X_BA_ = 26.9%, S_H2_ = 47.2% after 8 h of irradiation). TNT-400-Pt showed the highest selectivity toward propane (S_C3H8_ = 16.1%) with X_BA_ = 23.4% and S_H2_ = 36.2%. The activity results in conjunction with the characterization of the catalysts highlighted that the main factor affecting the activity in terms of X_BA_ and generation of H_2_ was the crystallinity, and in particular the presence of rutile phase in TiO_2_, whereas S_C3H8_ appears to increase when the electron-holes recombination is lower.

## Introduction

Nowadays, fossil fuels use is posing serious environmental issues (Raupach et al., 2007). Moreover, the global population is growing and global energy consumption is expected to increase. Thus, developing a process for energy production built on renewable resources is of utmost importance. Hydrogen (H_2_) is an energy carrier and an ideal fuel because it generates only water, and heat, which is in turn converted into electricity. With the development of fuel cells, the research on clean hydrogen production has found an additional application field. On the other hand, hydrogen is also a valuable chemical applied in several industrial processes.

Currently, 95% of the total hydrogen supply is provided from fossil fuels (Cargnello et al., 2011; Giddey et al., 2019). In the steam reforming process, methane (CH_4_) is converted into H_2_ at high temperature (typically 700 ÷ 900°C) (Halabi et al., 2010). This process is unsustainable on a long-term scale because of the limited availability of fossil fuels and the generation of greenhouse gases (mostly CO_2_). Consequently, many researchers are devoted to H_2_ production from alternative resources such as from biomass via catalytic steam reforming/gasification (Huber et al., 2003) and photoelectrochemical (Holladay et al., 2009) or enzymatic approaches (Lee et al., 2010). Most of these processes are often highly energy demanding owing to the strict operating conditions dictated by the use of enzymes (Cortright et al., 2011).

Solar energy, the largest renewable “infinite” resource, provides virtually unlimited energy to our planet (Lewis and Nocera, 2006). Therefore, the exploitation of sunlight is a very promising route in order to produce H_2_ and recent studies showed that it may be competitive with respect to traditional technology grounded on non-renewable sources (Rodriguez et al., 2014).

Photocatalytic H_2_ production from water is an interesting though challenging approach as it takes advantage of solar energy and operates under ambient pressure and temperature. The idea of using the photons in the solar spectrum to split H_2_O into H_2_ and O_2_ is known since 1972 (Fujishima and Honda, 1972). However, direct photosplitting of water has thermodynamic and kinetic limitations which can be overcome by adding oxygenated organic substrates, or “sacrificial agents,” which act as water reductants. This process, operating under anaerobic conditions, is called photoreforming (PR).

In the last decades, many researchers studied PR for H_2_ production. Several organic compounds were used as sacrificial agent [methanol (Maldonado et al., 2018), ethanol (Puga et al., 2014), glycerol (Vaiano et al., 2018), glucose (Iervolino et al., 2016), raw biomass (Granone et al., 2018), and 2-propanol (Tanaka et al., 2012) are some examples], while the most investigated photocatalysts were titanium dioxide and cadmium sulfide, properly designed on a case-by-case basis. Sacrificial agents with hydrogen atoms in the α-position with respect to hydroxyl group proved to be effective for H_2_ production since OH groups capture the photogenerated h^+^, thus mitigating the e^−^/h^+^ recombination (Bowker, 2012).

Butyric acid (BA) is a volatile fatty acid abundantly present in wastewater and is one of the main by-products of biological natural fermentation processes (Mu et al., 2006; Yu and Mu, 2006). This makes BA an inexpensive resource that can be converted to valuable chemicals such as small hydrocarbons and H_2_ through heterogeneous photocatalysis. BA has been very poorly studied as a sacrificial agent in PR. To the best of our knowledge, only two very recent studies covered this topic (Zheng et al., 2017; Li et al., 2018). Zheng and co-authors succeeded in the photodegradation of butyric acid under UV light using a composite made by Cu_2_O/Bi_2_WO_6_ and generating alkanes (mainly, methane, ethane and propane) and H_2_ (Zheng et al., 2017). Li et al. (2018) investigated photocatalytic H_2_ evolution from different organic fatty acids (OFA) under visible irradiation over carbon-doped TiO_2_ nanoparticles. In terms of H_2_ production rate, the observed order was: propionic acid > butyric acid ≈ acetic acid > lactic acid, at equal electron densities; and lactic acid > acetic acid > butyric acid ≈ propionic acid, at equal molar concentrations. They also found that mixed organic acids inhibited H_2_ generation, except in the case of acetic-lactic acid mixture, which led to an increase in the H_2_ evolution rate. The composition and distribution of the reaction products were not explored.

Moreover, photoreforming of butyric acid (BAPR) over TiO_2_ and noble metals (e.g., Pt, Au, Pd) has not been investigated yet. The valence band (VB) and conduction band (CB) of titania are bent when a noble metal is added because of the formation of a Schottky barrier, arising from the difference in the Fermi levels between the metal and the semiconductor (SC) (Christoforidis and Fornasiero, 2017). The higher is the work function (ϕ), the greater is the Schottky barrier in the metal-SC heterojunction, resulting in a more efficient charges separation, which is a critical step in most photocatalytic processes. Platinum, which has a very high work function (ϕ = 5.93 eV), generally showed better performance as TiO_2_-co-catalyst (Fu et al., 2008; Chiarello et al., 2010). Indeed, photogenerated electrons are more efficiently utilized on the platinum sites (Izumi et al., 1980).

In this work, BAPR over Pt-TiO_2_ based photocatalysts has been investigated for the first time, focusing on the products selectivity, along with the correlation of activity and selectivity to the optical, structural, textural and morphological properties of the catalysts used. Titanium oxide nanotubes were synthesized by hydrothermal method, calcined at different temperature (300, 400, 500°C) and decorated with platinum. Commercial P25 was used as reference sample. All the catalysts were tested in a bench scale reactor under UV light.

## Experimental

### Synthesis of TiO_2_ Nanotubes (TNT)

Commercial TiO_2_ (Aeroxide P25, 1.2 g) and 75 mL of a 10 M NaOH aqueous solution were placed in a 95 mL teflon lined hydrothermal autoclave reactor. After stirring and ultrasound treatment for 1 h, the reactor was kept in the oven at 110°C for 12 h. Next, the sample was poured into six 50 mL polypropylene conical tubes (BD Falcon), washed with DI water and separated from the supernatant through a centrifuge (at 8000 rpm, model Heraeus Megafuge 8R, from Thermo Scientific) several times, until the conductivity of the supernatant was below 70 μS/cm. Afterwards, the 6 tubes containing the powder were filled with a 0.1 M HCl aqueous solution, sonicated for 15 min and centrifuged once. Afterwards, the samples were washed with DI water (as described above) till the conductivity of the supernatant was around 2 μS/cm. The conductivity was measured by a Delta Ohm pH-χ-O_2_ meter (model HD 22569.2) and SPT 01 G probe (from Delta Ohm). The obtained powders were kept in oven at 80°C for 12 h and, eventually, grinded.

### Synthesis of TiO_2_ Calcined Nanotubes

TNT were calcined at different temperature in a furnace (Nabertherm P330) with the following temperature program: from 25°C to ${\text{T}}_{\text{calc}}^{{}^{\circ}}$C at 5°C/min, 3 h at T_calc_. The calcination temperatures (T_calc_) were: 300, 400, 500°C. These 3 samples are labeled as TNT-300, TNT-400, TNT-500.

### Platinum Photodeposition

Platinum (1 wt%) was photodeposited on all the samples (P25, TNT, TNT-300, TNT-400, TNT-500). First, H_2_PtCl_6_·6H_2_O was dissolved in ethanol in order to obtain a solution containing 0.5 mg of Pt per 1 mL ethanol. Next, 8 mL of that solution were added to 200 mL of distilled water in a beaker, mixed for 10 min, sonicated for 10 min, mixed for 10 min. Then, 400 mg of photocatalyst were added and this suspension was mixed for 5 min and sonicated for 10 min. Afterwards, argon was bubbled into the suspension in order to remove the dissolved oxygen and 75 min later, a UV LED lamp (placed on the top of the beaker) was switched on. After 4 h irradiation and continuous bubbling of argon, the suspension was collected, dried in a rotary evaporator (model RE300) under vacuum at 70°C, kept in oven at 70°C for 12 h and grinded. These samples are referred as P25-Pt, TNT-Pt, TNT-300-Pt, TNT-400-Pt, TNT-500-Pt.

### Photocatalyst Characterization

The crystalline structure of the samples was determined by an XRD PANalytical Empyrean diffractometer, a Cu Kα radiation of 1.54 Å, scan step-size 0.0167° and a 2θ scan range of 10–90°. The crystallite size was determined through the Scherrer equation with respect to the anatase and rutile major peaks at 25.33° and 27.50°, respectively (Howard et al., 1991; Swope et al., 1995), after the diffractogram background had been subtracted using the software HighScore Plus. In order to estimate the percentage of anatase and rutile in the TNTs, few XRD diffractograms were collected by mixing a certain photocatalyst with an equivalent mass (50/50 weight ratio) of CaF_2_ (Sigma-Aldrich, 99.9%). Before preparing such 50/50 weight ratio, TNT was kept 12 h at 85°C under vacuum whereas P25, P25-Pt, TNT-300, TNT-400, TNT-500 were kept at 160°C for 12 h under vacuum and CaF_2_ was kept at 200°C for 12 h. The ratio between the absolute crystallinity of sample and the absolute crystallinity of P25, for anatase (r_A_) and rutile (r_R_), were computed by applying equations reported by Jensen et al. (2004); the percentage of anatase in the crystalline phase was obtained by means of the Spurr and Myers equation (Spurr and Myers, 1957).

UV-vis diffuse reflectance spectra (DRS) have been collected by a UV/vis spectrophotometer (Shimadzu UV-2600) with an integrating sphere attachment and with BaSO_4_ as the reflectance standard.

The photoluminescence (PL) spectra in emission mode with an excitation at 300 nm were recorded using a Perkin Elmer LS 55 spectrometer between 320 and 580 nm (100 nm/min scan rate).

TEM and STEM-EDS analysis was performed by using Tecnai G2 and Titan FEI transmission electron microscopes, operating at 200 and 300 kV, respectively. The sample was prepared by suspending the powder in 2-propanol, ultrasounds treated, and finally dropping 5 uL of the suspension 3 consecutive times on a 400-mesh Cu grid provided by Tedpella, letting the solvent evaporate at room temperature.

Pore structure properties of the samples, including surface area, were obtained by means of a Flex Multiport Physisorption/Micropore Analyzer (Micromeritics, USA), using N_2_ as adsorbent. Before the analysis, the samples were degassed under vacuum at 80°C for 12 h. Surface area and total pore size distribution were obtained by applying Brunauer–Emmett–Teller (BET) theory. The microporosity was assessed through Horvath-Kawazoe model. The meso-pore volume was calculated by difference between total pore volume and micro-pore volume.

### Photocatalytic Runs

Photoreforming of BA was carried out in a Pyrex-made cylindrical batch photoreactor (total volume: 1090 mL) (see Supplementary Figure 1). The photoreactor was provided with valves in its upper section for the inlet and outlet of gases and for liquid and gas sampling. A 5 mM BA aqueous solution (temperature: 298 K; volume: 750 mL), containing 0.2 g/L of photocatalyst, was magnetically stirred and was bubbled with nitrogen 1 h before sealing the reactor by closing all the valves and switching the lamp on. The reactor was irradiated by 2 lamps made of LED strips (SMD 5050) with emission band between 360 and 370 nm. The lamps were placed outside the external walls of the reactor and inside a housing of Pyrex glass immersed in the reacting suspension and surrounded by a jacket where cooling water ensured a constant operational temperature and prevented LED strips from overheating. The absorbed electric power of the internal and external lamps were 7.2 and 12 W, respectively. The radiant power of both lamps was measured separately with a Delta Ohm 9721 radiometer in the ranges 315-400 nm: the external one was 6 W m^−2^ (measured at the bottom-center of the reactor) whereas the internal one was 2.28 W m^−2^ (measured at the external wall of the reactor). Each run was performed twice to confirm the reproducibility of the experiment.

Gas and liquid samples were withdrawn from the reactor at the beginning of and during the run. BA concentration was analyzed, after filtration of the liquid sample through a Whatman filter (0.2 μm TF), by means of a HPLC (UltiMate 3000, Thermo Scientific) equipped with a Phenomenex Rezex ROA—organic acid H^+^ (8%) column (size: 300 × 7.8 mm). The column was kept at 55 °C during the analysis and the eluent was a H_2_SO_4_ 0.0025 M aqueous solution at flow rate of 0.8 mL/min. Identification and quantification of the main products in gas phase were performed by 2 different gas chromatographs (GC's). The content of H_2_ and CO_2_ were measured by a Shimadzu GC-2014 equipped with thermal conductivity detector (TCD) and a micropacked ST MP-01 column (size: 2 m length, 1 mm ID; packing material: shincarbon ST); the temperature of injector, column and detector were kept at 230, 30, 100°C, respectively. Nitrogen was used as carrier gas at a flow rate of 6.81 mL/min.

The composition of alkanes in the gas sample were investigated by a Shimadzu GC-2014 equipped with a flame ionization detector (FID) and a capillary column (Agilent 19095P-S25PT, 50 m × 0.535 mm × 15μm); the temperature of injector and detector were kept at 200°C, 250, respectively. The initial temperature of the oven was 60°C for 5.5 min, followed by a ramp of 20°C/min to 100°C, an isotherm at 100°C for 21 min, a ramp 20°C/min to 140°C, and an isotherm at 140°C for 10 min. Helium was used as carrier gas with a flow rate of 6.13 mL/min. The experimental set-up has shown a reproducibility < 95% in terms of measured concentrations.

At the end of the run, the organic fraction of the liquid suspension was extracted by using diethyl ether in a separator funnel. Then, the organic solution was concentrated by evaporating the solvent, filtered and analyzed by a GC (Agilent Technologies 7890B) equipped with a quadrupole mass spectrometer (MS, Agilent Technologies 5977A MSD). The separation of the organic compounds was achieved through a HP-5ms ultra inert column (Agilent 19091S-433UI). The carrier gas was helium with a flow rate of 1 mL/min. The oven temperature was programmed as follows: initial temperature set at 50°C for 3 min, ramp from 50 to 100°C at 15°C/min, isothermal at 100°C for 5 min, ramp from 100 to 300°C at 10°C/min, isothermal at 300°C for 5 min. The MS transfer line was kept at 300°C. Mass spectra were collected in scan mode for qualitative analysis by comparison with the NIST mass spectral library. The software ‘NIST Mass Spectral Search Program' gives match factor and probability value. Match factor refers to the direct match between the unknown and the library spectrum. Probability value is derived assuming that the compound is represented by a spectrum in the libraries searched.

Total organic carbon (TOC) in the liquid sample was measured using a TOC-L (Shimadzu) analyzer. TOC conversion in liquid phase percent (X_TOC,liq_) was calculated as follows:

(1) $$\begin{array}{l} X_{TOC,liq}\;=\;\left[1-\left(\frac{TOC_{f}}{TOC_{0}}\right)\right]*\;100 \end{array}$$

where TOC_0_ and TOC_f_ are the TOC concentrations measured before and after irradiation, respectively.

The selectivity toward a compound i (S_i_) given in this paper has been computed as it follows:

(2) $$\begin{array}{l} S_{i}\;=\;\frac{N_{i}}{N_{BA}}*\text{​ }100 \end{array}$$

where N_i_ is the moles of i and N_BA_ is the reacted moles of BA, at the end of the reaction time (8h).

Details of the material balances discussed in this paper can be found in the Supplementary Materials.

## Results and Discussion

### Photocatalyst Characterization

**Figure 1:**
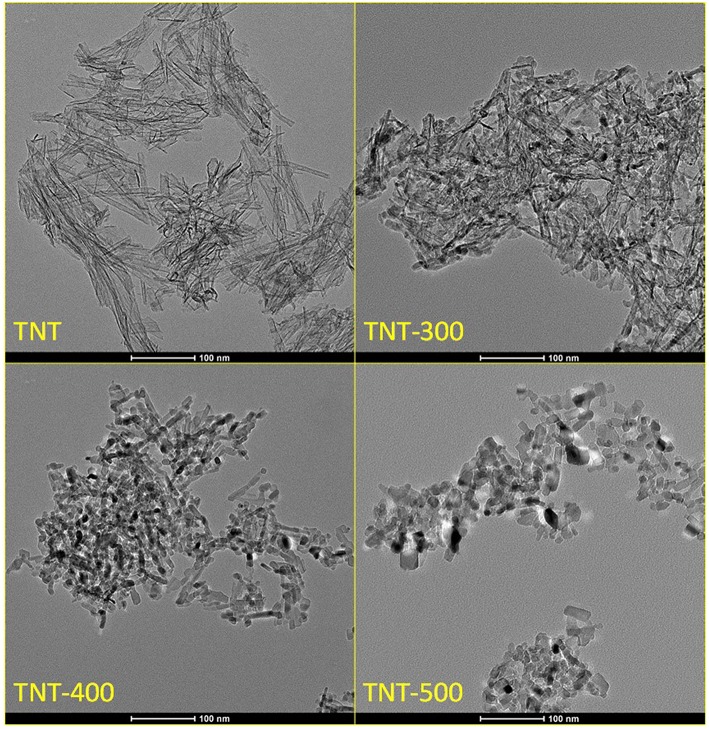
TEM images of TNT and TNT calcined at different temperature. Scale bar: 100 nm.

**Figure 2:**
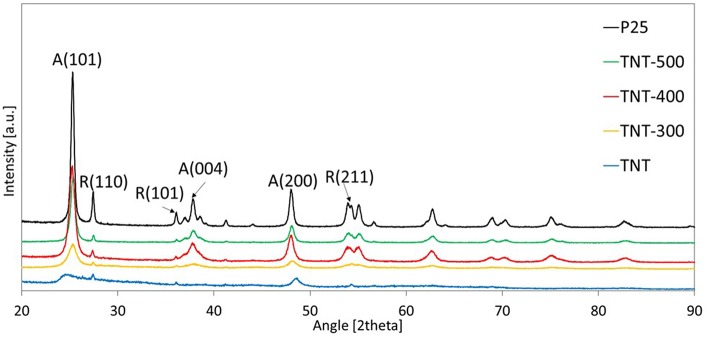
XRD patterns of different samples. The most important planes are indicated.

The external diameter, internal diameter and length of the synthesized TiO_2_ nanotubes (TNT) were in the range: 7–8.5, 4–5.5, 80–120 nm, respectively (Figure 1). XRD diffractogram of TNT showed that the predominant crystal planes are A(101), A(200) and R(110), R(101), R(211) for anatase and rutile allotropic phases of TiO_2_, respectively (Figure 2). The morphology as well as the crystallinity of TNT changed dramatically with the calcination temperature (T_calc_). When T_calc_ increases, the nanotubes evolve shrinking and shortening, eventually turning into a nanoparticle shape (Figure 1 and Supplementary Figure 2).

Table 1 reports the ratio between the absolute crystallinity of sample and the absolute crystallinity of P25, for anatase (r_A_) and rutile (r_R_), the percentage of anatase in the crystalline phase and the crystallite size of anatase and rutile. The size of anatase crystallite grows with T_calc_ from 3.1 nm (for TNT) to 15.5 nm (for TNT-500), whereas rutile crystallite size was the same in TNT and TNT-300, then it increased in TNT-400 and TNT-500. The absolute crystallinity of TNT was significantly lower compared to P25 (*r*_A_ = 12.2% and *r*_R_ = 8.5%). After calcination, all the previously missing crystal planes of anatase can be seen in XRD pattern (Figure 2). Indeed, r_A_ rose to 47.8% and 96.4% for TNT-300 and TNT-400, respectively; while r_R_ lowered for TNT-300. In other words, only new anatase phase is formed at 300°C. TNT-500 contained less anatase and more rutile than TNT-400, because at ca. 400°C the anatase to rutile phase transformation is already taking place (Hanaor and Sorrell, 2011). Nevertheless, it should be said that the weight fraction of rutile is relatively low for TNT, TNT-300 and TNT-400 (Table 1) and, for this reason, it is more difficult to estimate the variation of rutile among the samples. However, no rutile was formed at 300 °C because, with respect to TNT, r_R_ decreased and the crystallite size was the same (Table 1).

**Table 1:** Ratio between the absolute crystallinity of sample and the absolute crystallinity of P25 (r_A_, anatase; r_R_, rutile).

**Sample**	**r_**A**_ [%]^a^**	**r_**R**_ [%]^a^**	**Weight fraction anatase [%]^b^**	**Crystallite size anatase [nm]^c^**	**Crystallite size rutile [nm]^c^**
P25	100.0	100.0	89.0	22.8	34.4
TNT	12.2	8.5	92.1	3.1	13.4
TNT-300	47.8	2.9	99.2	8.3	13.7
TNT-400	96.4	3.9	99.5	12.9	17.8
TNT-500	94.6	21.1	97.3	15.5	18.5

Weight fraction of anatase in the crystalline phase of the sample. Crystallite size of anatase and rutile calculated by means of Scherrer's equation.

a Jensen et al. (2004).

b Spurr and Myers (1957).

c *Howard et al. (1991), Swope et al. (1995)*.

**Figure 3:**
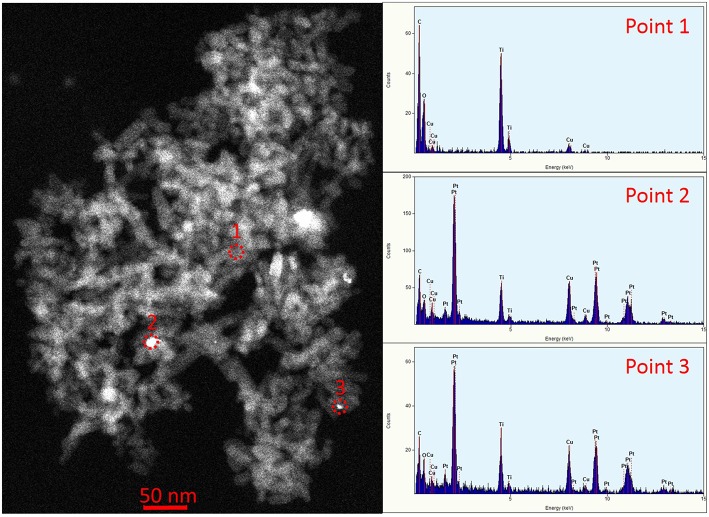
STEM image of TNT-500-Pt. EDS spectra correspond to points 1, 2, 3, in the image, respectively. The Cu peak is caused by the grid, whereas the C peak is caused by the grid and the sample.

The morphology of the TNTs did not change after the Pt-photodeposition (see Supplementary Figure 3). However, the STEM-EDS analysis corroborated the presence of Pt (agglomerate size < 15 nm) decorating TiO_2_ surface (Figure 3).

The textural properties of the samples are reported in Table 2. The Pt-photodeposition reduced the surface area of P25 and TNT. TNT-Pt surface area is one order of magnitude larger than P25-Pt. The surface area of TNT decreased with T_calc_ although it is still significantly larger than P25-Pt (Table 2).

**Table 2:** Textural properties of different samples.

**Sample**	**BET surface area [m^**2**^/g]**	**Micro-pore volume [cc/g]**	**Average micro-pore width [nm]**	**Meso-pore volume [cc/g]**	**Average pore diameter/width [nm]**	**Total pore volume [cc/g]**
P25	62.0	-	-	-	-	-
P25-Pt	53.0	-	-	-	26.86	0.222
TNT	359.0	-	-	-	-	-
TNT-Pt	297.5	0.138	0.926	0.898	13.75	1.036
TNT-300-Pt	208.7	0.087	1.133	0.778	16.26	0.865
TNT-400-Pt	128.2	0.054	1.110	0.198	7.86	0.252
TNT-500-Pt	86.1	0.036	1.127	0.355	17.77	0.391

**Figure 4:**
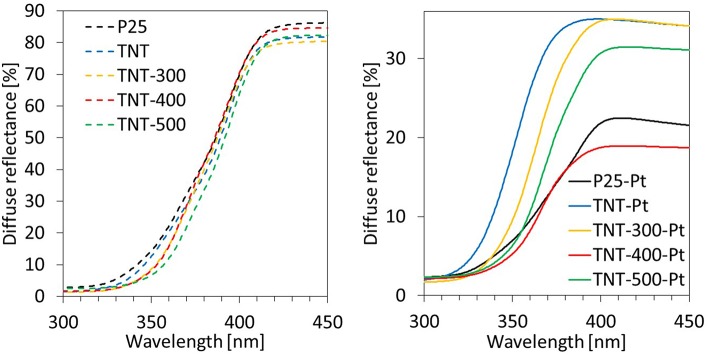
UV-vis diffuse reflectance spectra of different samples.

The optical properties of the samples have been investigated by recording UV-vis DRS (Figure 4). In the range of UV-A wavelengths, TNT and calcined TNT absorb more than P25 following the order P25 < TNT < TNT-300 ≈ TNT-400 < TNT-500. This trend can be partially explained by considering that defective states on the surface are introduced upon annealing, because of the transformation from amorphous to anatase phase, during calcination at 300°C, and then, from anatase to rutile phase during calcination at 500°C (Patel and Gajbhiye, 2012). In fact, absorption edge of TNT-300 and TNT-400 are comparable while it is higher for TNT-500.

**Figure 5:**
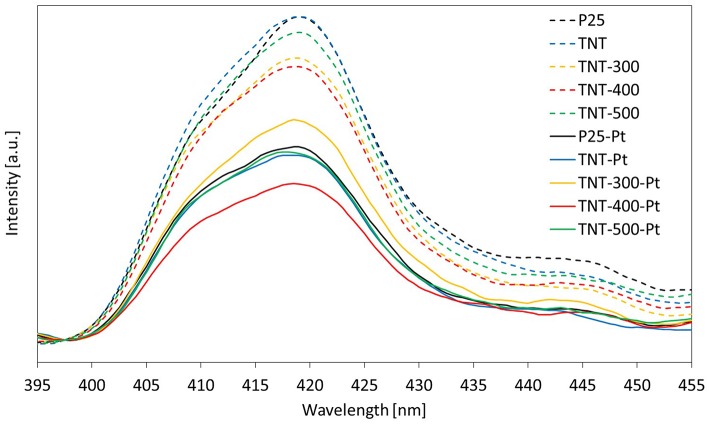
Photoluminescence spectra of different samples.

The broad emission peak about 400-435 nm in PL emission spectra (labeled as “PL peak,” Figure 5), upon excitation at 300 nm, is an indication of the extent of steady state e^−^/h^+^ recombination. The maximum intensity of the PL peak followed the order TNT-400 < TNT-300 < TNT-500 < TNT ≈ P25. Two factors are thought to affect this trend, which is different compared to the above DRS analysis: morphology and crystallinity of the sample. Calcination of TNT at 300°C and 400°C improved the e^−^/h^+^ separation efficiency because of higher percentage of crystalline phase than TNT (Table 1). However, the separation efficiency worsened in TNT-500 due to the absence of nano-sized structures (Figure 1).

Titania VB and CB are bent when Pt^0^ is deposited on the catalyst surface because of the formation of a Schottky barrier arising from the difference in the Fermi levels between the metal and the semiconductor (Christoforidis and Fornasiero, 2017). This event has implications on the DRS spectra. The absorption in the visible range (400–700 nm) increases because of the Pt. Nevertheless, photons in the visible range are energetically weak and they can result in the promotion of electrons from the VB of the semiconductor to intraband gap states which cannot result in the reduction of many substrates due to thermodynamic constraints. In addition, electrons photo-promoted from VB to CB of TiO_2_ can rapidly recombine with holes. Reactivity tests, discussed in the next section, provide important information about the efficiency of the light absorbed.

Focusing on the wavelength range 360–370 nm (the irradiation sources used in the reaction system emit mainly in the same range), the light absorbed by the samples with Pt was in this order: TNT-Pt < TNT-300-Pt < TNT-500-Pt < P25-Pt < TNT-400-Pt. This trend matched the percentage of absolute crystallinity (considering both anatase and rutile, Table 1): higher crystallinity, wider light absorption. However, TNT-400-Pt did not respect such correlation.

A noble metal such as Pt lowers the excitonic PL process intensity owing to the capture of metal ions and, therefore, enhancing e^−^/h^+^ separation during irradiation (Liqiang et al., 2006). Thus, the weaker the excitonic PL spectrum, the lower the recombination rate of photo-induced charge carriers.

As expected, all the samples showed a minor photoluminescence when compared to the equivalent ones without Pt (Figure 5) but, now, the maximum intensity of PL peaks order resulted as follows: TNT-400-Pt < TNT-Pt ≈ TNT-500-Pt < P25-Pt < TNT-300-Pt. The fact that the highest PL peak intensity among the samples with Pt was recorded for TNT-300-Pt was unexpected. A reasonable explanation could be that the surface area of TNT-300-Pt is lower than TNT-Pt and, consequently, the concentration of Pt per unit surface area increases (Table 2). TNT-400 surface area is even lower than TNT-300 but the crystallinity (Table 1) was higher, thus overall the e^−^/h^+^ recombination rate decreased. Exhaustive studies are required by taking into account morphology, crystallinity and Pt content of the sample at the same time, but this was not the main purpose of the present work.

To conclude this section, it is worth noting that the sample TNT-400-Pt exhibited the lowest e^−^/h^+^ recombination rate, as well as the highest light absorption in the range 350-370 nm.

### Photocatalytic Runs

No production of H_2_ as well as no significant degradation of BA took place in the absence of Pt as cocatalyst, confirming what is extensively reported in literature for other sacrificial agents implemented in similar reaction systems (Gallo et al., 2012; Languer et al., 2013; Al-Azri et al., 2015; Christoforidis and Fornasiero, 2017; Guayaquil-Sosa et al., 2017).

**Figure 6:**
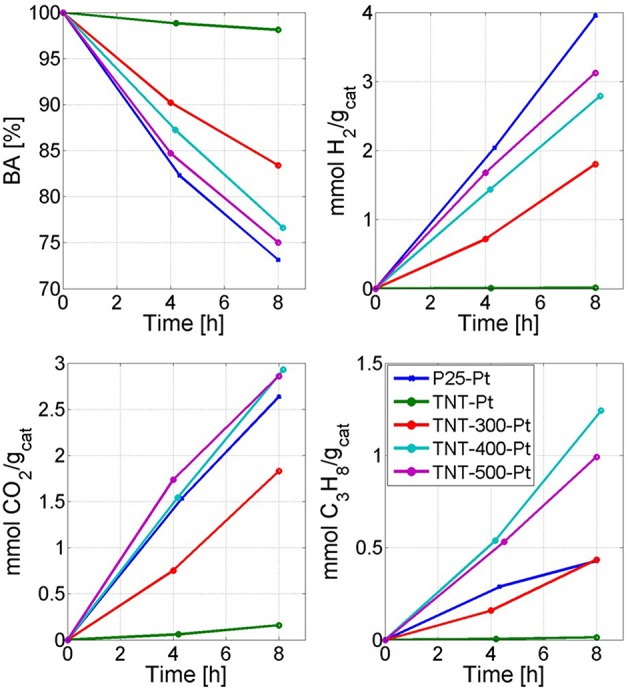
Reactivity results. Time refers to the irradiation time. The legend applies to all four plots.

The experimental results of BA photoreforming carried out in the batch photoreactor (Supplementary Figure 1) under UV light are shown in Figure 6 through 4 plots which report: BA conversion percentage (X_BA_), mmol of H_2_, propane (C_3_H_8_) and CO_2_ produced per gram of catalyst. In the Supplementary Figure 4, the peak area from the HPLC chromatogram of a significant unknown reaction by-product (ULP) is also shown. The whole following discussion refers repeatedly to Figure 6 and Table 3, unless otherwise stated.

**Table 3:** BA conversion (X_BA_), TOC conversion in liquid phase (X_TOC,liq_), selectivity toward H_2_ (S_H2_), selectivity toward propane (S_C3H8_), after 8 h of irradiation.

**Sample**	**X_**BA**_ [%]**	**X_**TOC, liq**_ [%]**	**X_**TOC, liq**_/X_**BA**_ [%]**	**S_**H2**_ [%]**	**S_**C3H8**_ [%]**
P25-Pt	26.9	14.0	52.1	47.2	5.1
TNT-Pt	1.9	0.3	13.5	0.6	0.6
TNT-300-Pt	16.6	6.9	41.5	29.5	7.1
TNT-400-Pt	23.4	18.9	80.8	36.2	16.1
TNT-500-Pt	25.0	18.7	74.8	30.0	11.8

In the run with P25-Pt, the conversion of BA following a zero order kinetics was equal to 26.9% after 8 h. The main reaction products in gas phase were H_2_, CO_2_ and C_3_H_8_, whereas other by-products, including methane and ethane, were detected by GC-FID. The amount of these gaseous by-products corresponded barely to 1% of the produced C_3_H_8_. There were also unknown by-products in liquid phase. Indeed, X_TOC, liq_ was half of X_BA_ after 8 h, even if it should be noted that part of the TOC_liq_ was converted to gas species (mainly C_3_H_8_).

When TNT-Pt was used, the BA degradation and the CO_2_ formation rates were around 15 times lower than P25-Pt, while the selectivity toward H_2_ and C_3_H_8_ (S_H2_, S_C3H8_) were almost negligible. This behavior of TNT-Pt can be ascribed to the low crystallinity (Table 1) and to absorption edge shifted to smaller wavelengths (Figure 4). Both drawbacks of the TNT were overcome by calcination at 300, 400 and 500°C. In terms of BA degradation and H_2_ evolution rates, none among TNT-300-Pt, TNT-400-Pt, TNT-500-Pt, performed better than P25-Pt, although both rates increased considerably when TNT samples were calcined. Remarkably, the S_C3H8_ was strongly affected by T_calc_. Indeed, BA conversion, H_2_ and CO_2_ production rates rose with T_calc_ in a monotonic way, while S_C3H8_ followed the order TNT-300-Pt < TNT-500-Pt < TNT-400-Pt.

It is remarkable that calcined TNTs (after Pt deposition) shifted the selectivity from some by-products, namely the amount of ULP decreased, toward C_3_H_8_ with respect to the run with P25-Pt. For instance, the production of C_3_H_8_ after 8 h were the same in runs with TNT-300-Pt and P25-Pt, even though X_BA_ was higher with in the latter case. This particular selectivity toward C_3_H_8_ became very evident at T_calc_ equal to 400 and 500°C when the production of C_3_H_8_ after 8 h increased by 190% and 132%, respectively, compared to P25-Pt (per unit catalyst mass). This dramatic change in the reaction products distribution was corroborated by a significant enhancement in X_TOC, liq_.

Finally, it should be mentioned that the surface area and X_BA_ followed exactly opposite trends. Thus, it can be assumed that the crystallinity plays a key-role in the photo-activity. For example, TNT-400-Pt surface area is larger than TNT-500-Pt, but the degradation of BA as well as the production of H_2_ were higher using TNT-500-Pt (Figure 6). However, as discussed above, the run with TNT-400-Pt highlighted S_H2_ and S_C3H8_ greater than the one with TNT-500-Pt (Table 3).

### Proposed Photocatalytic Mechanism

In the only previous study on BA photoreforming in which both the products distribution and the reaction mechanism were investigated (Zheng et al., 2017), the primary products were reported to be methane, propane, and ethane, with the last one being the most abundant alkane in the reaction system having either pure Bi_2_WO_6_ or pure Cu_2_O, or Bi_2_WO_6_/Cu_2_O composite as the photocatalyst. The results presented in this paper indicated that propane is the only significant gaseous product when powders of TiO_2_-Pt are used as photocatalyst in the BAPR. Thus, the photocatalytic mechanism must be different from the one previously published in literature, in which a drastically different catalyst was employed.

The formation of H_2_, CO_2_, C_3_H_8_, can be explained through the following possible photocatalytic mechanism (Equations 3–8):

(3) $$\begin{array}{lll} \text{Ti}{\text{O}}_{2}+\text{hν} & \to & \text{Ti}{\text{O}}_{2}\left({\text{e}}_{\left(\text{CB}\right)}^{-}+{\text{h}}_{\left(\text{VB}\right)}^{+}\right) \end{array}$$

(4) $$\begin{array}{lll} {\text{H}}_{2}\text{O}+{\text{h}}^{+} & \to & {\text{H}}^{+}+•\text{OH} \end{array}$$

(5) $$\begin{array}{lll} 2{\text{H}}^{+}+2{\text{e}}^{-} & \to & {\text{H}}_{2} \end{array}$$

(6) $$\begin{array}{lll} \text{C}{\text{H}}_{3}\text{C}{\text{H}}_{2}\text{C}{\text{H}}_{2}\text{COOH}+{\text{h}}^{+} & \to & \text{C}{\text{H}}_{3}\text{C}{\text{H}}_{2}\text{C}{\text{H}}_{2}\text{COO}•+{\text{H}}^{+} \end{array}$$

(7) $$\begin{array}{lll} \text{C}{\text{H}}_{3}\text{C}{\text{H}}_{2}\text{C}{\text{H}}_{2}\text{COO}• & \to & \text{C}{\text{H}}_{3}\text{C}{\text{H}}_{2}\text{C}{\text{H}}_{2}•+\text{C}{\text{O}}_{2} \end{array}$$

(8) $$\begin{array}{lll} \text{C}{\text{H}}_{3}\text{C}{\text{H}}_{2}\text{C}{\text{H}}_{2}•+{\text{H}}^{+}+{\text{e}}^{-} & \to & \text{C}{\text{H}}_{3}\text{C}{\text{H}}_{2}\text{C}{\text{H}}_{3} \end{array}$$

During the first stage of this mechanism (Equations 3–5), the photo-generated electrons and holes give rise to the water-splitting process over TiO_2_ surface (Wei et al., 2012), hydroxyl radical and H_2_ are two of the products at this stage. If no sacrificial agent is added, the hydroxyl radical would accumulate into the system hampering H_2_ evolution (Zheng et al., 2017). BA reacts on TiO_2_ surface according to Equation (6) to form the radical CH_3_CH_2_CH_2_COO• (Betts et al., 2018). Powders of TiO_2_-Pt act as a multitude of small, short-circuited Pt-TiO_2_ electrode systems. Platinum particles distributed over titania surface lower the otherwise significant overpotential of hydrogen reduction (Kraeutler and Bard, 1978). The e^−^/h^+^ separation is boosted because of Equations (5) and (6), which occur on 2 different site (Pt and TiO_2_). Then, the radical CH_3_CH_2_CH_2_COO• decomposes quickly to CH_3_CH_2_CH_2_• and CO_2_ (Equation 7). Propane is formed through Equation (8). According to Kraeutler and Bard, alkanes are primarily produced by using H atom of the carboxylic group (Kraeutler and Bard, 1978).

**Figure 7:**
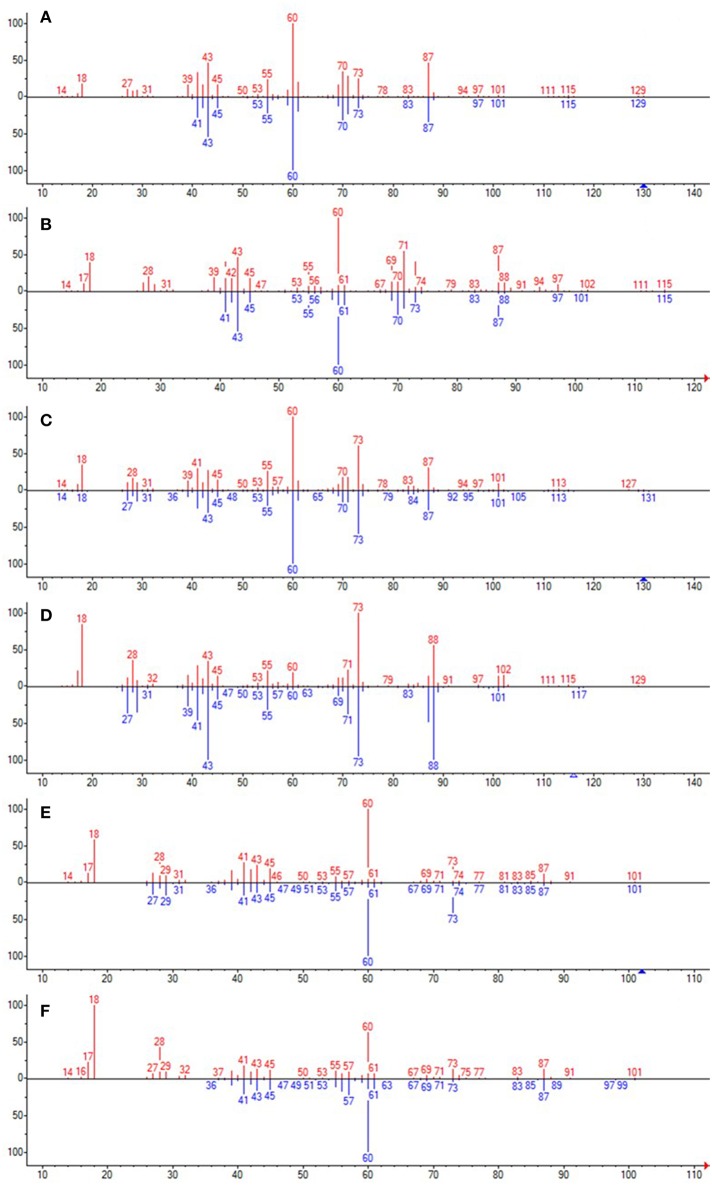
The unknown mass spectrum from GC-MS analysis (in red) associated with the library spectrum (in blue) for the following compounds: 3-methylhexanoic acid (both stereoisomers, **A,B**), heptanoic acid **(C)**, 2-ethylbutanoic acid **(D)**, pentanoic acid **(E)**, 3-methylpentanoic acid **(F)**.

Analysis of the liquid products by means of GC-MS suggested the compounds shown in Table 4 (MS spectra in Figure 7).

**Table 4:** Compounds detected by means of GC-MS during the analysis of liquid samples.

**Name**	**Formula**	**Structure**	**Retention time [min]**	**Match factor [%]**	**Probability value [%]**	**Eq**.
3-Methylhexanoic acid	C_7_H_14_O_2_	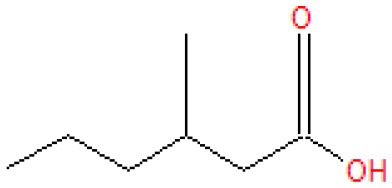	7.91	92.6	92.3	9
3-Methylhexanoic acid	C_7_H_14_O_2_	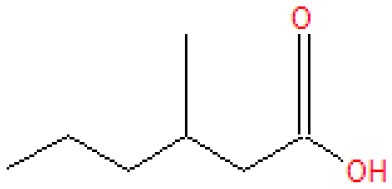	8.03	80.9	68.3	9
Heptanoic acid	C_7_H_14_O_2_	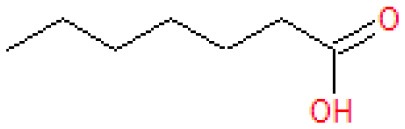	8.62	89.3	79.6	10
2-Ethylbutanoic acid	C_6_H_12_O_2_	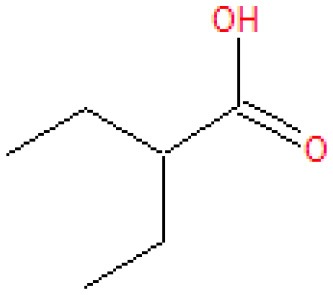	7.51	80.5	61.0	11
Pentanoic acid	C_5_H_10_O_2_	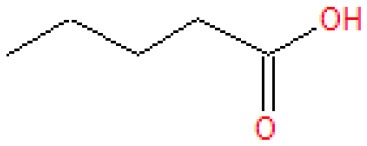	5.43	83.4	54.7	12
3-Methylpentanoic acid	C_6_H_12_O_2_	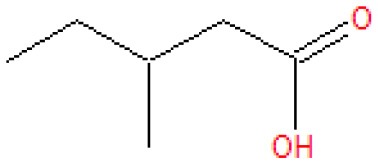	6.25	83.9	67.1	13, 14

*Note that 3-methylhexanoic acid has 2 stereoisomers with 2 different retention times in the GC-MS chromatogram*.

These possible products could be generated through Equations (9–14).

(9) $$\begin{array}{lll} \text{C}{\text{H}}_{3}\text{C}{\text{H}}_{2}\text{C}{\text{H}}_{2}•+\text{C}{\text{H}}_{3}\text{C}{\text{H}}_{2}\text{C}{\text{H}}_{2}\text{COOH} & \to & \text{C}{\text{H}}_{3}\left(\text{C}{\text{H}}_{3}\text{C}{\text{H}}_{2}\text{C}{\text{H}}_{2}\right)\text{CHC}{\text{H}}_{2}\text{COOH}+\text{H}• \end{array}$$

(10) $$\begin{array}{lll} \text{C}{\text{H}}_{3}\text{C}{\text{H}}_{2}\text{C}{\text{H}}_{2}•+\text{C}{\text{H}}_{3}\text{C}{\text{H}}_{2}\text{C}{\text{H}}_{2}\text{COOH} & \to & \text{C}{\text{H}}_{3}\text{C}{\text{H}}_{2}\text{C}{\text{H}}_{2}\text{C}{\text{H}}_{2}\text{C}{\text{H}}_{2}\text{C}{\text{H}}_{2}\text{COOH}+\text{H}• \end{array}$$

(11) $$\begin{array}{lll} \text{C}{\text{H}}_{3}\text{C}{\text{H}}_{2}\text{C}{\text{H}}_{2}•+\text{C}{\text{H}}_{3}\text{C}{\text{H}}_{2}\text{C}{\text{H}}_{2}\text{COOH} & \to & \text{C}{\text{H}}_{3}\text{C}{\text{H}}_{2}\left(\text{C}{\text{H}}_{3}\text{C}{\text{H}}_{2}\right)\text{CHCOOH}+\text{C}{\text{H}}_{3}• \end{array}$$

(12) $$\begin{array}{lll} \text{C}{\text{H}}_{3}•+\text{C}{\text{H}}_{3}\text{C}{\text{H}}_{2}\text{C}{\text{H}}_{2}\text{COOH} & \to & \text{C}{\text{H}}_{3}\text{C}{\text{H}}_{2}\text{C}{\text{H}}_{2}\text{C}{\text{H}}_{2}\text{COOH}+\text{H}• \end{array}$$

(13) $$\begin{array}{lll} \text{C}{\text{H}}_{3}\text{C}{\text{H}}_{2}\text{C}{\text{H}}_{2}•+\text{C}{\text{H}}_{3}\text{C}{\text{H}}_{2}\text{C}{\text{H}}_{2}\text{COOH} & \to & \text{C}{\text{H}}_{3}\left(\text{C}{\text{H}}_{3}\text{C}{\text{H}}_{2}\right)\text{CHC}{\text{H}}_{2}\text{COOH}+\text{C}{\text{H}}_{3}• \end{array}$$

(14) $$\begin{array}{lll} \text{C}{\text{H}}_{3}•+\text{C}{\text{H}}_{3}\text{C}{\text{H}}_{2}\text{C}{\text{H}}_{2}\text{C}{\text{H}}_{2}\text{COOH} & \to & \text{C}{\text{H}}_{3}\left(\text{C}{\text{H}}_{3}\text{C}{\text{H}}_{2}\right)\text{CHC}{\text{H}}_{2}\text{COOH}+\text{C}{\text{H}}_{3}• \end{array}$$

When the propyl radical does not react with an e^−^/h^+^ pair (Equation 8), it triggers radical reactions with BA (Equations 9–13). Several products are generated (Table 4), depending on which position of BA the propyl radical attack takes place. Two different peaks in the GC-MS chromatograms resulted to be assignable to 3-methylhexanoic acid with a high probability, suggesting that both stereoisomers were formed. 3-Methylpentanoic acid could be formed directly from BA (Equation 13) or from pentanoic acid (Equation 14).

The traces of methane and ethane, which were shown to be formed in the previous section, could be due to the following reactions:

(15) $$\begin{array}{lll} \text{C}{\text{H}}_{3}•+{\text{H}}^{+}+{\text{e}}^{-} & \to & \text{C}{\text{H}}_{4} \end{array}$$

(16) $$\begin{array}{lll} \text{C}{\text{H}}_{3}•+\text{C}{\text{H}}_{3}• & \to & \text{C}{\text{H}}_{3}\text{C}{\text{H}}_{3} \end{array}$$

As mentioned above, this photocatalytic mechanism (Equations 3–16) has been hypothesized in order to explain the formation of the identified products. However, many other reactions can take place, especially when radical species are present in the reaction system. The unidentified species (ULP), which appeared in the HPLC chromatograms, could be one of the compounds in Table 4. The formation of alkenes cannot be ruled out since reactions as Equation 17, for instance, are possible under anaerobic conditions.

(17) $$\begin{array}{l} \text{C}{\text{H}}_{3}\text{C}{\text{H}}_{2}\text{C}{\text{H}}_{2}•+{\text{h}}^{+}\to \text{C}{\text{H}}_{3}\text{CHC}{\text{H}}_{2}+{\text{H}}^{+} \end{array}$$

The selectivities S_H2_ and S_C3H8_ depend on the fate of h^+^ and e^−^. They can react over Pt generating H_2_ or over TiO_2_ by forming C_3_H_8_. In addition, charge carriers recombination is fundamental at this stage. P25-Pt, and TNT-400-Pt have similar DRS spectra (Figure 4) but the latter sample revealed the lowest e^−^/h^+^ recombination rate (Figure 5). TNT-400-Pt showed a lower figure of S_H2_ but higher S_C3H8_ (Table 3), when compared to P25-Pt. Several considerations should be mentioned here. First, the more C_3_H_8_ is formed through Equation (8), the less electrons are available for H_2_ evolution (Equation 5); also, 2 electrons and 2 protons are required to produce form hydrogen (Equation 5) while only one electron and one proton (and a propyl radical) are required to form C_3_H_8_ (Equation 8). Then, when the propyl radical is involved in Equations (9–11, 13), S_C3H8_ decreases, while more liquid by-products are formed (low X_TOC, liq_/X_BA_ ratio, Table 3).

### Material and Electron Balances

In order to further investigate the products, detailed carbon and electron balances were conducted over the batch reactor, based on the consumed BA (100% of C or e^−^) and products after 8 h irradiation (see Table 5). The number of electrons equivalents refer to the degree of reduction, namely to the number of electrons an electron donor (or acceptor) can donate (or accept) per mole of compound per unit of carbon (γ), in order for it to be completely oxidized (or reduced) to reference compounds (Shuler et al., 2017). These material balances aim to estimate the carbon fraction that ended up in products other than CO_2_, CH_4_, C_2_H_6_, C_3_H_8_, and in which phase (liquid or gaseous) such fraction is present. The selectivity appears clear toward liquid products (Table 4) rather than gaseous ones, when P25-Pt was used. In fact, the fraction of unknown products in liquid (C_UL_) and gaseous (C_UG_) phase, in terms of C-mol%, were 58.5 and 11 %, respectively. On the contrary, with the calcined TNT (TNT-300-Pt, TNT-400-Pt, TNT-500-Pt), C_UL_ decreased and C_UG_ increased (see Table 5). The last results confirmed that calcined TNT are more selective toward gaseous products such as alkanes (and probably alkenes). Overall, the percentage of total C as unknown products (C_UT_) was in the range from 52.1% (in the case of TNT-400-Pt) to 69.5% (with P25-Pt).

**Table 5:** Carbon and electron balance after 8 h of irradiation.

		**P25-Pt**	**TNT-300-Pt**	**TNT-400-Pt**	**TNT-500-Pt**
C as butyric acid (reactant)	mmol C	3.80	2.28	3.40	3.81
	%	100.0	100.0	100.0	100.0
C as methane+ethane+propane+CO_2_ in gaseous phase (C_KG_)	mmol C	0.56	0.45	0.96	0.84
	%	14.8	19.9	28.3	22.0
Dissolved CO_2_ (C_KL_)	mmol C	0.60	0.42	0.67	0.63
	%	15.7	18.3	19.7	16.5
Total C as known product (C_KT_)	mmol C	1.16	0.87	1.63	1.47
	%	30.5	38.2	47.9	38.5
C as unknown products in liquid phase (C_UL_)	mmol C	2.22	0.83	1.02	0.51
	%	58.5	36.5	30.0	13.3
C as unknown products in gaseous phase (C_UG_)	mmol C	0.42	0.58	0.75	1.84
	%	11.0	25.3	22.1	48.2
Total C as unknown products (C_UT_)	mmol C	2.64	1.41	1.77	2.34
	%	69.5	61.8	52.1	61.5
e^−^ as butyric acid (reactant)	mmol e^−^	19.01	11.40	17.01	19.05
	%	100.0	100.0	100.0	100.0
e^−^ as methane+ethane+propane+H_2_ (gaseous phase)	mmol e^−^	2.37	1.79	4.41	3.76
	%	12.5	15.7	25.9	19.8
e^−^ as other products (gaseous and liquid phase, ${\text{e}}_{\text{UT}}^{-}$)	mmol e^−^	16.64	9.61	12.60	15.28
	%	87.5	84.3	74.1	80.2
γ = ${\text{e}}_{\text{UT}}^{-}$/ C_UT_	mmol e^−^/mmol C	6.30	6.82	7.12	6.52

*Note that C_KT_ = C_KL_+C_KG_; C_UT_ = C_UL_+C_UG_; C_KT_+C_UT_ = 100%; γ, degree of reduction per atom C*.

The electron balances (shown in Table 5) indicate that the percentage of e^−^ transferred from BA to unidentified compounds (${\text{e}}_{\text{UT}}^{-}$, including those in Table 4) ranged between 74.1% (TNT-400-PT) and 87.5% (P25-Pt).

The degree of reduction of the compounds in Table 4 is between 5.20 and 5.43. The ratio ${\text{e}}_{\text{UT}}^{-}$/C_UT_ (in mmol/mmol) gives an average γ of the unidentified reaction products (Table 5), which is in the range 6.30–7.12. This results suggest that other possible products in gaseous phase (or dissolved in liquid phase) could be linear and branched alkanes such as butane, pentane, 2-methylbutane, 2-methylpentane, which are yielded by decarboxylation of other species (see Table 4), because they have γ = 6.33 ÷ 6.50.

## Outlook and Conclusions

In this study the photoreforming of butyric acid under UV light was studied by applying both commercial and modified TiO_2_ in a batch reaction system. The main identified reaction products were H_2_, CO_2_, propane and organic acids with more carbon atom than BA (e.g., pentanoic and 3-methylhexanoic acid). The presence of platinum (1 wt%) in TiO_2_ was essential for PR to take place.

The different morphology and crystallinity of the samples affected dramatically their optical properties, and hence, the reaction rate of PR and the products distribution. The highest conversion of BA and selectivity toward H_2_ were recorded with P25-Pt (X_BA_ = 26.9%, S_H2_ = 47.2% after 8 h). TiO_2_ nanotubes calcined at 400°C, after Pt photodeposition, showed the highest selectivity toward propane (S_C3H8_ = 16.1%) with still important figures for both BA conversion and S_H2_ evolution (X_BA_ = 23.4%, S_H2_ = 36.2%).

The experimental results described in this work prove that production of valuable chemicals besides H_2_ is possible from butyric acid. Also several factors, such as morphology, crystallinity, charge carrier recombination, light absorption, and cocatalyst, appear to affect the activity and selectivity of the reaction. Currently, photocatalytic generation of H_2_ is not applied at industrial level since the small production rates make it unappealing. However, the coproduction of H_2_ and propane from an inexpensive abundant resource such as BA could boost the attractiveness of this process, promoting its future scale-up.

TiO_2_-Pt based catalysts are possible candidates for BAPR. However, the efficiency in terms of conversion and selectivity to target products can certainly be improved. For instance, designing photocatalysts active under both UV and visible light is still a challenge, especially for scale-up application. Narrowing the SC band gap by doping (Leung et al., 2010; Taylor et al., 2014; Sun et al., 2015) and incorporating visible light sensitizers [e.g., organic dyes (Manfredi et al., 2016), transition metal complexes (Duonghong et al., 1981) or another SC (Park et al., 2008; Wang et al., 2013)] are the two main strategies for attaining that goal. Indeed, oxidation of aromatic alcohols with H_2_ production was obtained via electron transfer from Pt nanoparticles, active under visible light, to the TiO_2_ CB (Zhai et al., 2011).

BAPR is proven to be able to upgrade low value butyric acid into a portfolio of higher value products. The average degree of reduction estimated for the large fraction of unknown products suggests a composition of high value either as chemicals or fuels.

In summary, efficient charge separation and absorption at wavelength > 400 nm are two main features that should be further investigated in order to make photoreforming of butyric acid a process suitable for scale-up application. An economic evaluation of photoreforming process will be also very relevant.

## Data Availability

All datasets generated for this study are included in the manuscript/Supplementary Files.

## Author Contributions

GS performed the experimental activities and drafted the article. JR contributed to the data analysis and the mass balances. GP coordinated the research activities and contributed to the writing of the article. All the authors contributed to the research idea.

### Conflict of Interest Statement

The authors declare that the research was conducted in the absence of any commercial or financial relationships that could be construed as a potential conflict of interest.
