# Supplementary material for: Hydrogen and Propane Production From Butyric Acid Photoreforming Over Pt-TiO2
Source: Front Chem. 2019 Aug 7;7:563. doi: 10.3389/fchem.2019.00563 (PMC6692698; doi:10.3389/fchem.2019.00563)
Supplement: Supplementary file 1 [file Data_Sheet_1.pdf]

Supplementary Material

Hydrogen and propane production from butyric acid photoreforming over Pt-TiO_2

Gabriele Scandura[1,2], Jorge Rodríguez[1], Giovanni Palmisano[1,2*]

[1]Department of Chemical Engineering, Khalifa University, Masdar Institute Campus , PO Box 54224, Abu Dhabi, United Arab Emirates.

[2]Research and Innovation Center on CO_2 and H_2 (RICH), Khalifa University, P.O. Box 127788, Abu Dhabi, United Arab Emirates.

*** Correspondence:**
Giovanni Palmisano
giovanni.palmisano@ku.ac.ae

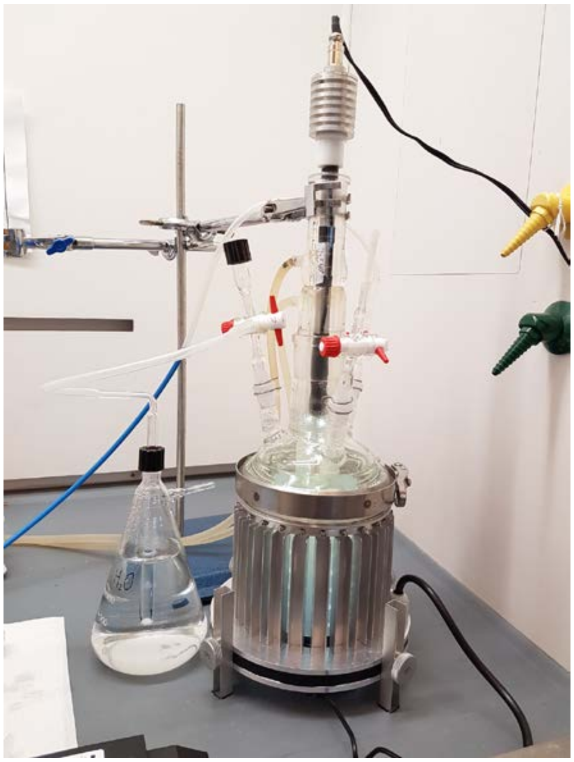

Supplementary Figure 1. Picture of the reaction system used for the photocatalytic runs.

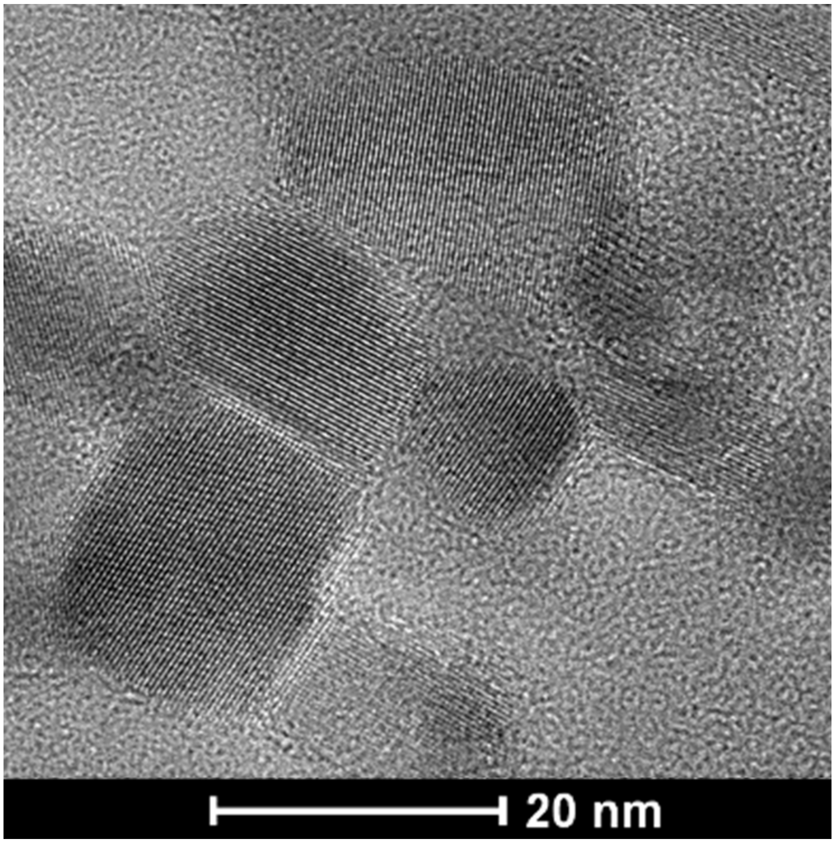

Supplementary Figure 2. TEM image at high magnification of TNT-400.

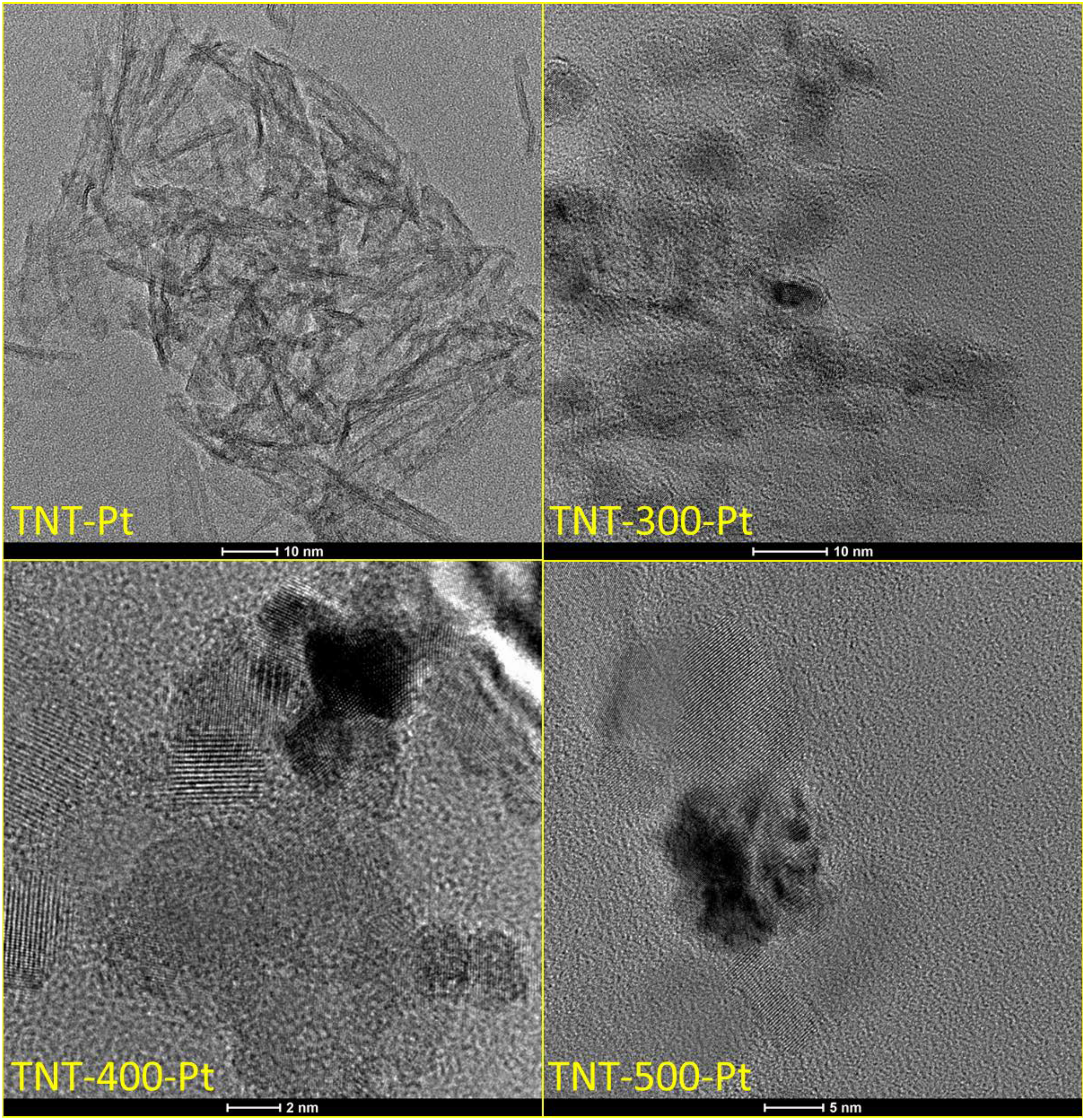

Supplementary Figure 3. TEM images of TNT and TNT calcined at different temperature and decorated with platinum.

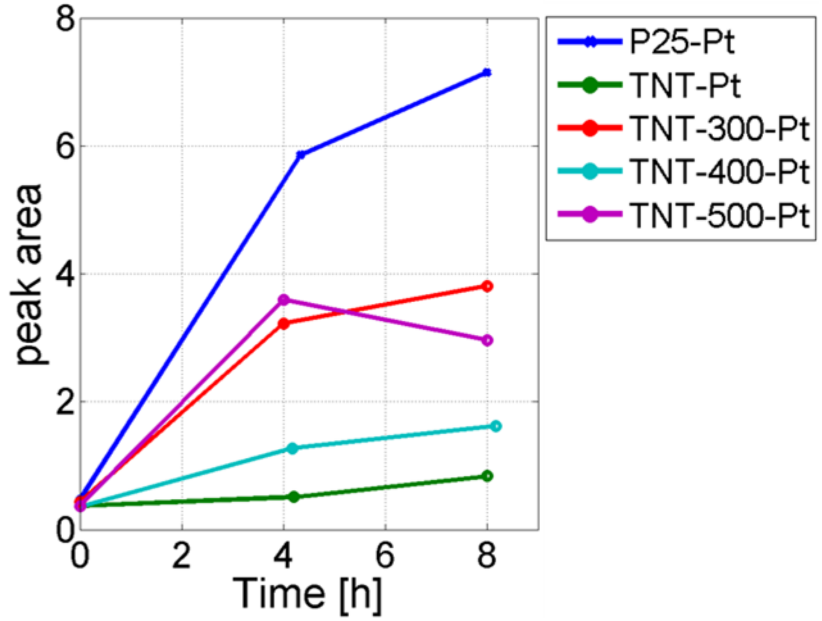

Supplementary Figure 4. Peak area from the HPLC chromatogram of a significant unknown reaction by-product.

Material balances details

The concentration of methane ($[CH_4]$), ethane ($[C_2H_6]$), propane ($[C_3H_8]$) and CO_2 in gas phase were obtained through GC analysis and multiplied by the total gas volume in the reactor. Similarly, the concentration of butyric acid ([BA]), given by HPLC, was multiplied by the total liquid volume in the reactor. The amount of CO_2 dissolved in water ($[CO_2]_L$) was calculated by means of Henry's law, knowing the concentration of CO_2 in the reactor headspace ($[CO_2]_G$), the room temperature and the pH of the solution (3.61).

The quantities in table 5 (in the manuscript) were computed as follows:

$C_{BA,R} = ([BA]_0-[BA]_f)*V_L*4$

$TOC_R = (TOC_0-TOC_f)*V_L/AW_C$

$C_{BA,R} = C_{KT} + C_{UT}$

$C_{KT} = C_{KL} + C_{KG}$

$C_{UT} = C_{UL} + C_{UG}$

$C_{KL} = [CO_2]_L$

$C_{KG} = ([CH_4] + [C_2H_6]*2 + [C_3H_8]*3 + [CO_2]_G)*V_G$

$C_{UL} = TOC_{BA,R} – TOC_R$

$C_{UG} = TOC_R – C_{KG} – [CO_2]_L$

where:

AW_C = carbon atomic weight

V_L = total liquid volume in the reactor

V_G = total gas volume in the reactor

$C_{BA,R}$ = carbon as BA reacted (mol C)

TOC_0 = initial total organic carbon in liquid phase (mg/L)

TOC_f = final total organic carbon in liquid phase (mg/L)

TOC_R = TOC reacted (mol C)

C_{KT} = total carbon as known product (mol C)

C_{UT} = total carbon as unknown products (mol C)

C_{KL} = carbon known products in liquid phase (mol C)

C_{KG} = carbon as methane+ethane+propane+CO_2 in gaseous phase (mol C)

C_{UL} = carbon as unknown products in liquid phase (mol C)

C_{UG} = carbon as unknown products in gaseous phase (mol C)

C_{KT}, C_{KL}, C_{KG}, C_{UT} , C_{UL}, C_{UG} can also be given as fraction (%) of $C_{BA,R}$.
